# Peripherally Administered Nanoparticles Target Monocytic Myeloid Cells, Secondary Lymphoid Organs and Tumors in Mice

**DOI:** 10.1371/journal.pone.0061646

**Published:** 2013-04-23

**Authors:** Iraklis C. Kourtis, Sachiko Hirosue, Alexandre de Titta, Stephan Kontos, Toon Stegmann, Jeffrey A. Hubbell, Melody A. Swartz

**Affiliations:** 1 Institute of Bioengineering, École Polytechnique Fédérale de Lausanne, Lausanne, Switzerland; 2 Swiss Institute for Experimental Cancer Research (ISREC), École Polytechnique Fédérale de Lausanne, Lausanne, Switzerland; 3 Institute of Chemical Sciences and Engineering, École Polytechnique Fédérale de Lausanne, Lausanne, Switzerland; 4 Mymetics BV, Leiden, The Netherlands; National University of Ireland, Ireland

## Abstract

Nanoparticles have been extensively developed for therapeutic and diagnostic applications. While the focus of nanoparticle trafficking *in vivo* has traditionally been on drug delivery and organ-level biodistribution and clearance, recent work in cancer biology and infectious disease suggests that targeting different cells within a given organ can substantially affect the quality of the immunological response. Here, we examine the cell-level biodistribution kinetics after administering ultrasmall Pluronic-stabilized poly(propylene sulfide) nanoparticles in the mouse. These nanoparticles depend on lymphatic drainage to reach the lymph nodes and blood, and then enter the spleen rather than the liver, where they interact with monocytes, macrophages and myeloid dendritic cells. They were more readily taken up into lymphatics after intradermal (i.d.) compared to intramuscular administration, leading to ∼50% increased bioavailability in blood. When administered i.d., their distribution favored antigen-presenting cells, with especially strong targeting to myeloid cells. In tumor-bearing mice, the monocytic and the polymorphonuclear myeloid-derived suppressor cell compartments were efficiently and preferentially targeted, rendering this nanoparticulate formulation potentially useful for reversing the highly suppressive activity of these cells in the tumor stroma.

## Introduction

Nanosized particles have found various applications, such as in drug delivery [1], *in vivo* imaging contrast agents [2], and in vaccination [3]. Nanoparticulate drug formulations can behave differently from their soluble counterparts due to size-dependent lymphatic drainage and uptake pathways [4]. Their tunable parameters, including size as well as core and surface chemistries, may be tailored to fit a desired application, and they can further be modified with bioactive ligands, such as antibody fragments or homing peptides, to enhance their targeting capabilities [1], [5], [6]. Such platforms exploit the specificity of the covalently bound ligands to carry their payload to target cells and tissues for therapeutic or diagnostic applications [7]. Such applications span from simple passive carriers and solubilizers for hydrophobic drugs, such as chemotherapeutics, to even more complex systems that specifically trigger immune responses for vaccines [8]–[11]. In all of these applications, the distribution of nanoparticles after administration is an important question, including understanding toxicity concerns [12].

We and others have previously shown that by exploiting lymphatic drainage, sub-100 nm particles drain freely to the lymph nodes (LNs) after injection in lymphovascularized tissues such as the dermis [10], [13]; the nanoparticles are sufficiently small as to not be entrapped in the network structure of the tissue interstitium, and the interstitial flow convects them into the draining lymphatic vessels. Exploiting size permits local LN targeting without the use of specific targeting ligands [14] or direct injection into LNs [15]. In the LN, the nanoparticles are rapidly taken up by antigen presenting cells (APCs) and can therefore be used to modulate immune responses [10], [16], [17].

In nature, viruses range in size from ∼25 to 200 nm and traffic to the draining LN [18] where, depending on their glycosylation pattern and targeting of specific cell subsets [19], they are transported to the blood, spleen and liver to elicit different immunological outcomes. Similarly, vaccine delivery platforms [3], [10] are often engineered to be in this size range, because draining LNs can guide the lymph to cell specific compartments [20]. As nanosized particulates engage the same entrance and clearance mechanisms as viruses [3], and as the cell type these agents first encounter has an impact on infectious and immunological outcomes [18], we were motivated to investigate the exact cellular distribution followed by the fully synthetic Pluronic-stabilized poly(propylene sulfide) (PPS) nanoparticles (NPs, using this abbreviation to refer only to these specific nanoparticles) that we have previously described and used as a vaccine platform [8]–[10], [16], [21].

## Materials and Methods

### Mice

C57Bl/6 female mice, 6–8 wks old, were purchased from Harlan Laboratories (Gannat, France). K14-VEGFR-3-Ig transgenic mice [22], which lack dermal lymphatic drainage, were a kind gift of Dr. Kari Alitalo (University of Helsinki, Finland). All experiments were performed with the permission of the Veterinary Authority of the Canton de Vaud, Switzerland. Immunizations were performed under isofluorane.

### Ethics statement

All mouse procedures and manipulations were conducted according to the ethical principles and guidelines for housing and experiments on animals of the Swiss federal veterinary office. The animal protocol (protocol number 2235) describing the procedures was reviewed and approved by the veterinary affair office of the canton of Vaud, Switzerland.

### Chemicals, reagents and antibodies

All chemicals were reagent grade and purchased from Sigma-Aldrich (Saint Louis, MO) unless otherwise noted. Antibodies for flow cytometry were purchased from eBioscience (San Diego, CA) and BioLegend (San Diego, CA) unless otherwise noted. Cell viability was determined using either propidium iodide (PI) or Live/Dead cell dyes (Invitrogen, Zug, Switzerland). For intracellular staining, cells were fixed and permeabilized using BD Cytofix/Cytoperm Fixation/Permeabilization Solution Kit (BD Biosciences, San Jose, CA). Secondary antibodies for immunofluorescence were from Invitrogen.

### Nanoparticle synthesis

Pluronic-stabilized poly(propylene sulfide) nanoparticles (referred to herein simply as NPs) were synthesized as previously described [23]. Briefly, 5.07 mmol of propylene sulfide (400 µL) was emulsified in 5% (w/v) Pluronic F127 (500 mg) in 10 mL of ddH_2_O. In-house synthesized four-arm initiator (40.2 µmol, 14.8 mg) was used to start the polymerization of propylene sulfide. After dialyzing for 48 h, 2 mL of NPs (30 mg/mL) were fluorescently labeled with 0.048 µM (0.05 mg) of Dy649-maleimide (NP-Dy649) maleimide (Dyomics GmbH, Jena, Germany) in phosphate buffered saline (PBS) at RT for 24 h, followed by gel filtration in a Sepharose CL6B size exclusion gravity column with PBS (150 mM) as eluent [23] or dialyzed against 0.9% NaCl. Fractions containing the most fluorescent NPs were pooled together. The presence of free dye was assessed by size exclusion HPLC chromatography (Figure S1). NP biotinylation was accomplished by reacting derivatized Pluronic F127 carboxyl [23] with 5 equivalents of N-(+)-Biotinyl-3-aminopropylammonium trifluoroacetate salt and 10 eq. of N-hydroxysuccinimide and 1-Ethyl-3-(3-dimethylaminopropyl) carbodiimide overnight. Biotinylated NPs were dialyzed prior to use.

### Virosome production

To produce a fluorescent lipid (L-α-phosphatidylethanolamine-Dy649), 200 nmol L-α-phosphatidylethanolamine (Avanti Polar Lipids, Birmingham, AL) was added to 200 nmol Dy649-NHS (Dyomics GmbH, Jena, Germany) in 200 µL methanol, and the reaction was carried out in the dark, under argon gas, at room temperature. After 40 min, 3.3 nmol of triethylamine were added, and the reaction was terminated after 80 min. The reaction product and free dye were separated by TLC as above, and re-extracted with methanol; the yield of the coupling reaction at this point was 36.6% (as determined by absorption at 649 nm). Preparative full-scale HPTLC of the reaction product followed by repeated (3×) recrystallization from methanol yielded 17.8 nmol of L-α-phosphatidylethanolamine-Dy649 as determined by phosphate analysis.

Approximately 5 mg of A/Singapore/6/86 influenza virus (1.5 µmol of membrane phospholipid) was inactivated by 0.05% beta-propiolactone (Molekula, Dorset, UK) overnight at 4°C, pelleted by centrifugation, and dissolved in 1,2-dicaproyl-s-*n*-phosphatidylcholine (DCPC; Avanti Polar Lipids, Birmingham, AL); the nucleocapsid was then removed by centrifugation. To the supernatant, 750 nmol of phospholipid 22.5 nmol of L-α-phosphatidylethanolamine-Dy649, dissolved in 100 mM DCPC in HNE buffer (145 mM NaCl, 5 mM HEPES, 1 mM EDTA), was added. The resulting mixture was sterilized by filtration through 0.22 µm, and dialyzed extensively against HNE in gamma-irradiated slide-a-lyzers (Pierce, Rockford, IL US, 30 kD MW cut-off).

### Particle characterization

#### Fluorescence Characterization

Fluorescence intensity was normalized by taking a known weight of NPs and measuring the particle suspension fluorescence using a plate spectrometer (Saphire, Tecan Group Ltd., Männedorf, Switzerland).

#### Endotoxin Characterization

All of the evaluated formulations were tested for endotoxin levels prior to use (<0.01 EU/mg) utilizing the TLR4 HEK Blue system (Invivogen, Carlsbad, CA US) using an LPS standard (Sigma-Aldrich, Taufkirchen, Germany).

#### Size Characterization

NP size measurements were obtained by dynamic light scattering (DLS) on a Nano ZS Zetasizer instrument (Malvern Instruments, Malvern, UK) with a 632 nm laser using PMMA cuvettes. NP solutions were diluted with PBS (150 mM pH 7.4) to a final NP concentration of 2 mg/mL before measurement. Particle sizes (in nm) were obtained as the *Z*-average by fitting the correlation function using the cumulant method. Dy649-labeled particles were measured in dilute dispersions on a Brookhaven goniometer system (Brookhaven Ltd, Worcestershire, UK) using an Lexel-Laser argon laser light source operating at a wavelength of 488 nm at a fixed detector angle of 90°C. Fluorescence of NP and VS were normalized by their fluorescence of Dy649 in the presence of 0.1% Triton X100, as measured in a fluorescence plate reader.

### Bioavailability study

Mice were administered either 1.8 mg of NP-Dy649 (15 mg/mL) intravenously (i.v.), or 450 µg in each footpad i.d. or intramuscularly (i.m.). At various timepoints (10 min, 1 h, 6 h, 12 h, 24 h, 48 h, 96 h and 144 h), 5 µL of blood was withdrawn from the tail and immediately diluted 40× in HBSS (pH = 7.4). The fluorescence of blood samples was measured in a plate reader (excitation 655 nm, emission 676 nm) and correlated with a standard curve of NP-Dy649 in whole blood. Bioavailability 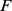 was determined according to the following ratio: 
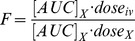
, where AUC is the area under the curve and X is i.d. or i.m., respectively.

### Nanoparticle Cellular Distribution

Mice were inoculated with 450 µg (15 mg/mL) of NP-Dy649 per footpad. Blood was drawn from each mouse and leukocytes were isolated using a 60% Percoll step gradient after 20 min centrifugation at 800× g at room temperature. Organs (the lungs, liver, kidneys, skin draining LNs and spleen) were harvested after transcardial perfusion with PBS. Tissue was gently dissociated and incubated in Collagenase D (Roche Ltd., Mannhein, Germany) for 45 min at 37°C and 5% CO_2_. Single-cell suspensions were acquired after passing the digested organs through a 70 µm strainer. Lungs, liver, and kidneys leukocytes were isolated using a 40%, 70% Percoll step gradient as described above. Cells were stained with the following panels: (1) I/Ab-FITC, F4/80-PE, CD11b-PerCP5.5, CD11c-Pacific Blue, CD8α-eFluor750, GR-1 biotin and streptavidin Pacific Orange, and (2) CD25-FITC, TCRγδ-PE, B220-PerCP5.5, CD4-PeCy7, CD3ε-Pacific Blue, CD8α-eFluor750, CD45-biotin & streptavidin Pacific Orange. Flow cytometry was performed with a CyAn ADP analyzer (Beckman Coulter, Nyon, Switzerland).

### Intradermal versus intramuscular administration

Mice were administered with 450 µg (15 mg/mL) of NP-Dy649 per limb, injected i.d. or i.m.. After 1, 6, 12, 24 or 144 h, LNs and spleens were harvested. As above, tissue was processed to single cell suspensions, stained, and analyzed by flow cytometry.

### Immunofluorescence

Organs were harvested and immediately frozen in Tissue-Tek® O.C.T. Compound (Sakura, Torrence, CA) in isopentane and liquid N_2_, and sectioned at 9 µm (LNs) or 40 µm (spleens). Sections were post-fixed in acetone at −20°C. LN sections were either stained for hematoxylin and eosin, or immunostained for (i) CD35 (biotinylated rabbit anti-mouse, clone 8C12, BD Pharmingen, Franklin Lakes, NJ US) with streptavidin-A488 (Invitrogen) and DAPI, or (ii) Lyve-1 (rabbit anti-mouse, 103-PA50AG, ReliaTech, Wolfenbüttel, Germany) and ER-TR7 (rat anti-human/mouse, HM1086, Hycult biotech, Uden, The Netherlands) with secondary antibodies, A488-rabbit anti-mouse IgG and A546-goat anti-rat,IgG, respectively (Invitrogen). Images were taken on a Zeiss LSM 710 confocal microscope (Carl Zeiss Inc., Oberkochen, Germany) and in an upright Leica DM5500 (Leica Microsystems, Bensheim, Germany). Images were processed on ImageJ (v1.45b, NIH, Bethesda, MD).

### Endocytosis assessment using flow cytometry

Splenocytes from naïve C57Bl/6 female mice were isolated as described above. Single-cell suspensions were re-suspended in RPMI 1640 medium supplemented with 10% FBS, 1% puromycin/streptomycin (all from Invitrogen) and plated in 96 round well plates (BD) to a final volume of 200 µL. Cells were treated with 225 µg of either biotinylated NPs or control NPs (without biotin) and were incubated at 37°C, 5% CO_2_ overnight. The following morning cells were washed twice with chilled HBSS and stained with Live/Dead Fixable Aqua (Invitrogen) for 20 min on ice. Cells were washed with HBSS, 0.1% BSA and stained for extracellular markers (CD45, B220, CD4, CD8α or CD11b, Ly6c and Ly6g) and streptavidin A488 (1∶500) in HBSS, 0.1% BSA, for 20 min on ice. Stained cells were washed with HBSS and fixed with 2% PFA for 15 min on ice. Afterwards, cells were washed twice with HBSS, 0.1% BSA, 0.5% Saponin (permeabilization buffer) and stained with streptavidin-A647 solution (1∶500) in permeabilization buffer for 30 min on ice. Finally cells were washed once with permeabilization buffer and re-suspended in 200 µL HBSS, 0.1% BSA for flow cytometry.

### Data analysis and visualization

All flow cytometry data were analyzed using FlowJo software (v9.2, Treestar Inc., Ashland, OR). The gating strategy to identify the major cellular subsets is depicted in Figures S2 and S3. NP negative gates were set on cells derived from animals treated with non-fluorescent NPs. Heat maps, which show median values of the percentages of NP^+^ cell types at each timepoint and organ, were generated using custom software in MATLAB (v7.11, Mathworks Inc., Natick, MA). Assuming that the cellular NP association or uptake is independent for each cell type, each cellular population, was modeled with a Beta regression model using the *betareg* package [24] within R (v2.13.1 R Foundation for Statistical Computing) with *logit* as the link function for the means submodel and *square root* link function for the variable precision submodel. The mean response (% of NP^+^) was modeled as a combination of the main effects and interactions of the route of administration, time post inoculation and organ of interest, while the variable precision as the combination of the main effects of the route of administration, time post inoculation and organ of interest, respectively. The full model was submitted according to the formula:
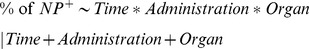
The contribution of each factor was calculated as the log-likelihood ratio (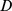) of the likelihood of each nested model (i.e the full model without one of the factors) over the likelihood of the full model: 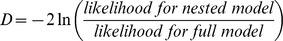. The significance of each alternate model versus the full model was computed using a *Chi* Square test. Statistical analysis was performed in Graphpad Prism (v5.0c, Graphpad Software, Inc, La Jolla, CA US) where appropriate.

### Tumors

EL-4 lymphoma cells stably transfected with OVA (E.G7-OVA cells, CRL-2113, ATCC, Manassas, VA) were cultured in RPMI 1640 medium supplemented with 10% FBS, 10 mM HEPES, 1 mM sodium pyruvate, 0.05 mM β-mercaptoethanol, 1% puromycin/streptomycin (all from Invitrogen), and 0.4 mg/mL G418 (PAA Laboratories GmbH, Pasching Austria). B16-F10 melanoma cells (CRL-6475, ATCC, Manassas, VA US) were cultured in high glucose (4.5 g/L) DMEM (PAA Laboratories) supplemented with 10% FBS, L-glutamine and 1 mM sodium pyruvate (Invitrogen). Both cell lines were washed twice with saline prior to inoculation. For the E.G7-OVA tumor model, mice were anesthetized with isofluorane, shaved on the left shoulder (dorsoanterior left lateral side), and inoculated subcutaneously with 10^6^ E.G7-OVA cells under the left shoulder blade. For the B16-F10 model, mice were anesthetized with isofluorane, shaved on the back, and inoculated subcutaneously with 5×10^5^ B16-F10 cells. Tumor volumes were estimated after measuring length (*l*), width (*w*), and height (*h*), and assuming an ellipsoid geometry (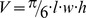). When tumor volumes reached 100 mm^2^, mice were administered i.d. with fluorescently labeled NPs in all four footpads.

## Results and Discussion

### Nanoparticles accumulate in the blood and secondary lymphoid compartments in antigen presenting cells

**Figure 1:**
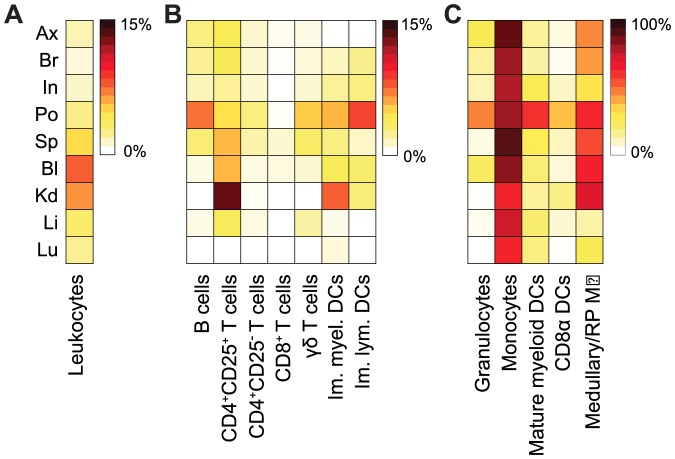
Nanoparticle biodistribution in tissues and cells show secondary lymphoid organ accumulation. Heat maps show nanoparticle (NP)-positive percentages of each indicated cell type in lymph nodes (LN) or blood-filtering organs 12 h after i.d. injection as analyzed by flow cytometry. (**a**) Overall leukocyte (CD45+) in different tissues. leukocyte subpopulations with (**b**) low to medium levels (0–15%) or (**c**) high levels (up to 98%) of NP accumulation. B cells: B220^+^, T cells: (CD3ε^+^ then CD4^+^CD25^+^, CD4^+^CD25^−^, CD8^+^), TCRγδ: CD3ε^+^CD4^−^CD8^−^ TCRγδ^+^, immature myeloid dendritic cells (DCs): CD11c^+^CD11b^+^I/A^b−^, immature lymphoid DCs: CD11c^+^CD11b^−^I/A^b−^. (**c**) Granulocytes: CD11b^+^GR1^high^SSC^high^, monocytes: CD11b^+^GR1^low^SSC^low^F4/80^+^, mature myeloid DCs: CD11c^+^CD11b^+^I/A^b+^, CD11c^+^CD8α^+^I/A^b+^, CD11c^+^CD11b^−^I/A^b+^, medullary macrophages (MØ): CD11b^+^F4/80^+^. Draining LNs are indicated by Ax: axillary, Br: brachial, In: inguinal, Po: popliteal; Sp: spleen, Bl: blood, Kd: kidneys, Li: liver, Lu: lungs. Heatmap color scales indicated on the right. Refer to gating strategies in Figures S2 and S3.

We evaluated the cellular distribution of 30 nm NPs 12 h after i.d. footpad administration in all leukocytes (Figure 1a), further dissecting the NP association using major markers within the innate and adaptive immune compartments (Figures 1b and c, with gating strategies shown in Figures S2 and S3). With regard to leukocyte localization in different anatomical locations (Figure 1a), NP^+^ leukocytes were found at high levels in the blood, kidneys and spleen (8±3%, 6±2% and 3±1%, respectively; Figure 1a). This distribution can be attributed partially to the poly(ethylene glycol)-rich (PEGylated) corona and to their ultra-small size, which has been demonstrated to bias NPs towards the spleen rather than the liver [25].

We next considered which leukocyte compartments were differentially targeted in the draining LNs, blood, and blood-filtering organs (liver, kidney and spleen) (Figure 1b, c). Consistent with lymphatic uptake after i.d. administration, NP uptake in the draining LNs was observed, with the popliteal LN (the sentinel LN, i.e. the LN that is closer to the hindlimb injection site than inguinal LN, or the axillary or brachial are to the forelimb injection site) collecting the injected NPs most efficiently. In the draining LNs, NPs associated at high levels with immature lymphoid dendritic cells (DCs, CD11c^+^CD11b^−^I/Ab^−^; 8.5±19.9%) and B cells (B220^+^; 7.3±5.6%), as well as other phagocytic cell populations, such as the immature myeloid DCs (CD11c^+^CD11b^+^I/A^b−^; 5.7±19.8%), cross-presenting DCs (CD11c^+^CD8α^+^I/Ab^+^; 36±16%) and medullary (LN) or red pulp (spleen) macrophages (CD11c^+^CD11b^+^F4/80^+^I/Ab^−^; 70±20%).

Those NPs that passed through the lymphatic circulation entered the bloodstream through the thoracic duct. In the blood, circulating NPs populated the entire compartment of circulating (blood) and resident (LNs and spleen) monocytes (CD11b^+^GR1^+^SSC^low^F4/80^+^).

Once in the blood, the NPs were subject to clearance in the blood-filtering organs. The spleen retained a similar cell association pattern as the blood, dominated by monocytes and medullary/red pulp macrophages, with the pattern less intensively followed in the liver and lungs (Figure 1c). In contrast, NP association patterns with cell types in the kidney were distinctly different from those of the spleen, liver, and lungs. In particular, NPs were associated strongly with activated (CD25^+^) CD4^+^ T cells in the kidneys (10±9%) (Figure 1b); although the reasons for this are unclear. Strikingly, throughout all the organs evaluated, NPs were found to be predominantly cleared by the mononuclear phagocytic system (MPS) after 12 h (Figure 1c).

These results were particularly interesting for immunomodulatory targeting, including for vaccination. Macrophage subsets have different roles in vaccination, where subcapsular macrophages form a primary barrier against the spread of infectious agents [18] and furthermore start the antigen transfer chain towards follicular dendritic cells for effective B cell responses [26], [27]. Medullary macrophages, which were very effectively targeted by the NPs in the draining LNs as well as the red pulp macrophages in the spleen, also form a clearance mechanism for particles that enter these organs [28], although less efficient than DCs for T cell priming [29]. DC-specific targeting, which is more interesting for vaccination [30]–[32], was most effective in the draining LNs and spleen. Both immature myeloid and lymphoid DCs have migratory capacities and can process antigens to prime T cell responses [33]. Mature myeloid DCs, migrating under steady-state conditions, may derive from the periphery [34], [35]. At 12 h post i.d. immunization, we see these populations positive for NPs, especially in the popliteal LN. Moreover, cross-presenting DCs (CD11c^+^CD8α^+^I/Ab^+^) resident in the LNs as well as the spleen were targeted; this cell population is particularly interesting for vaccine applications in that it is capable of priming CD8^+^ T cell responses [36] by loading exogenous antigen, e.g. nanoparticle-bound antigen, onto MHC I. This observation is consistent with our previous studies of MHC I-binding peptide antigens conjugated to NPs inducing strong expansion of the antigen-specific CD8^+^ T cell compartment [8], indicating that cross-presentation of the antigen had taken place.

The ability to target B and T lymphocytes in the LNs and spleen may be of value in immunomodulation. Direct modulation of B and T cells, if more efficiently targeted, may be of interest in the *in vivo* implementation of the auto-feedback of T cell activation by particles cross-linked to T cells previously shown for *ex-vivo* activation and adoptive transfer [11].

As a point of comparison with biological particles, we examined the biodistribution of virosomes derived from A/Singapore/6/86 influenza virus, which have been shown to produce effective antibody titers in clinical trials [37]. In stark contrast to the NPs, virosomes were cleared predominantly in the liver and were associated with T cells there (Figure S4). They also targeted, although to a much lesser extent, monocytes, medullary macrophages, and immature lymphoid and myeloid DCs in the LNs (Figure S4). Such predominantly hepatic uptake of the virosomes has been documented earlier [38], while the NPs largely avoided clearance by the liver and accumulation in the spleen. This was likely due to the smaller NP size (<50 nm) and PEGylated corona [25]. It is well established that biomaterials such as PEG attribute “stealth-like” characteristics and therefore ensure long-circulation; they are opsonized by the MPS as they trigger the alternative complement pathway [39]. Most of the studies that address the biodistribution of fully or partially synthetic particulates focus either at the organ level [40] or examine only the major immune cell populations [13], [16]. While earlier studies have reported that nanoparticles are selectively cleared by the MPS [16], our studies of APC subtype distributions show that NPs have a unique and distinct association pattern, with a tendency to accumulate in cells of myeloid origin in lymphoid tissues rather than in the lymphocyte populations of the liver.

### Intradermal nanoparticle administration favors rapid myeloid cell accumulation

**Figure 2:**
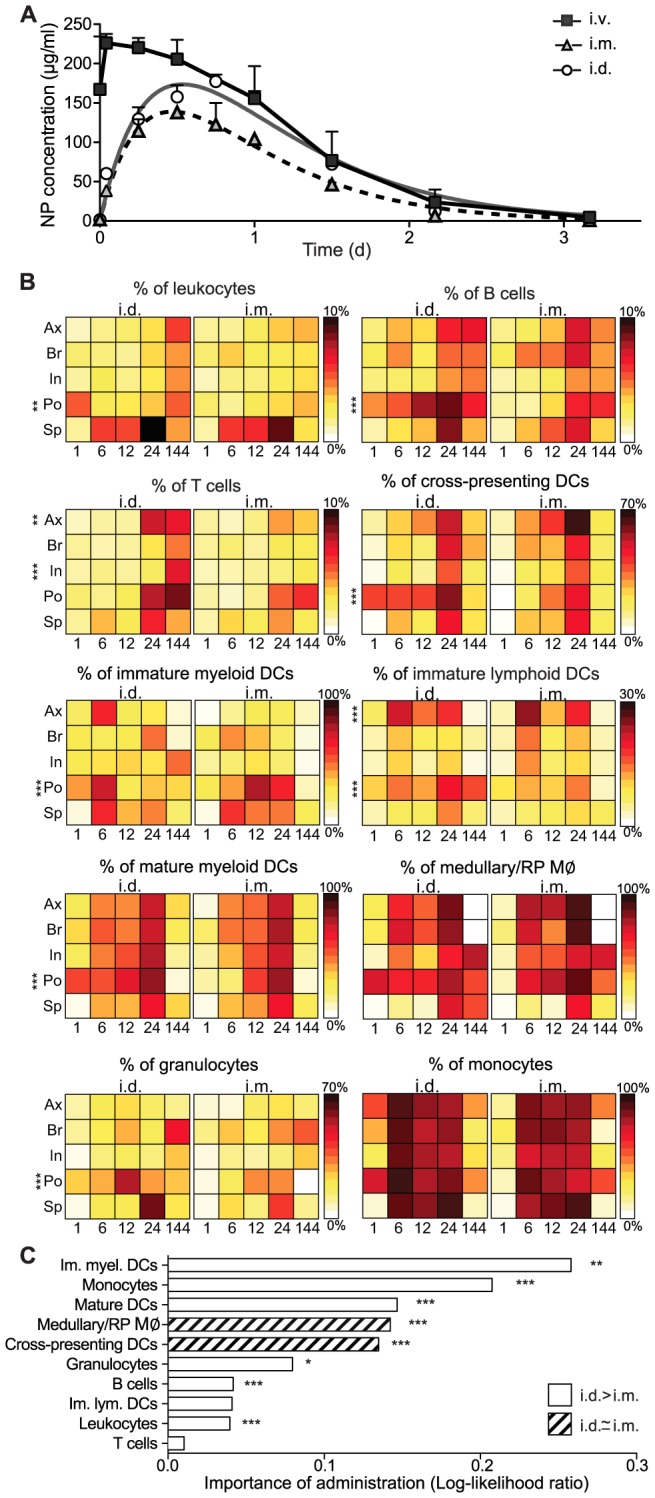
Nanoparticles target lymph node dendritic cells better after i.d. vs. i.m. delivery. (**a**) Blood concentrations of Dy649-labeled NPs after i.v., i.m. and i.d. administration. (**b**) Heat maps representing the median percentage of NP^+^ cells for indicated cell populations. Note that maxima vary from 10% in total leukocytes to 100% in monocytes. *P* values were computed by comparing the adjusted means of each organ between i.d. and i.m. for each cell type with a two-tailed Student's t-test. (**c**) Importance of route of administration for each cellular subtype. The log-likelihood ratio represents the likelihood of the alternate model, i.e. the model without taking account the route of administration, over the likelihood of the full factorial model. *P* values were computed using the *Chi* Square test between the alternate model and the full model for each population. For 144 h, *n* = 2, for all else, *n*≥4. Leukocytes: CD45^+^, mature myeloid DCs: CD11c^+^CD11b^+^I/A^b+^, cross-presenting DCs: CD11c^+^CD8α^+^ I/A^b+^, immature myeloid DCs: CD11c^+^CD11b^+^I/A^b−^, immature lymphoid DCs: CD11c^+^CD11b^−^I/A^b−^, medullary/red pulp (RP) macrophages (MØ): CD11b^+^F4/80^+^, monocytes: CD11b^+^GR1^mid^SSC^low^F4/80^+^, granulocytes: CD11b^+^GR1^high^SSC^high^, T cells: CD3ε^+^, B cells: B220^+^. Draining lymph nodes are indicated by Ax: axillary, Br: brachial, In: inguinal, Po: popliteal; Sp: spleen. *p≤0.05, **p<0.01, ***p<0.005.

While previous studies have compared immune responses to various vaccination routes, especially with more recent i.d. delivery technologies such as microneedles [41], it is still unclear how i.d. vs. i.m. routes of administration influence the efficiency of nanoparticulate delivery to various cell compartments as well as subsequent immune responses [3], [40], [42]. We observed that i.m. administration resulted in lower bioavailability in blood as compared to i.d. (54% vs. 83%, respectively; Figure 2a). We then compared NP kinetics and cellular distribution in the blood and the secondary lymphoid organs after i.d. vs. i.m. administration (Figure 2b). Overall, NPs administered i.d., but not i.m., extensively associated with leukocytes in the popliteal LN within the first hour (5±3% vs. 2±4% respectively in the popliteal LN); in the other LNs, peak NP association was observed after 6 d (Figure 2b first panel). B and T cells showed peak association after 24 h for both routes of administration, and both compartments showed stronger association after i.d. administration compared to i.m. Since T and B cells accounted for the majority of the leukocytes within the LNs, the higher percentages of NP^+^ leukocytes on day 6 was attributed mainly to T cells.

In comparing APC subsets, as was seen in Figure 1c, major populations of the MPS were found to be positive for NPs. Consistent with the overall leukocyte association, i.d. delivery resulted in early associations in sentinel skin-draining LNs nearly regardless of the cell population studied. Throughout the first 12 h post-inoculation (and only in the popliteal LN), the CD8α^+^ DCs that are notable for their efficient cross-presentation capacity [43], [44] internalized NPs to a higher extent after i.d. administration (1 h: 40±10%, 6 h: 40±20%, 12 h: 40±20%) than after i.m. (1 h: 1±1%, 6 h: 20±20%, 12 h: 30±20%). In the initial time points (6 h) after i.d. administration, immature myeloid and lymphoid DCs showed enhanced NP uptake within the axillary LN (60±30% and 20±20% respectively) and popliteal LN (80±20% and 14±2% respectively), while accumulation was pronounced only after 12 h of i.m. administration (80±30%, 10±20%). The peak uptake of mature myeloid DCs was not affected by the route of administration, reaching a maximum 80±10% for both i.d. and i.m. administration in the popliteal node (Figure 2b).

Within the window of 12–24 h after injection, nearly all of the medullary macrophages were associated with NPs in the draining LNs nodes irrespectively of the administration route (Figure 2b, 8^th^ panel). This is consistent with the mode of NP entry in the LN, where smaller nanoparticles flow to the LN thus associating with medullary APCs [13]. Granulocytes were minimally affected by the route of administration, with the pattern of association being less intense and delayed than for the other cells of the MPS. Most strikingly, monocytes were NP^+^ in all organs, including the spleen, as early as 6 h and until 24 h. However, at 6 days, monocytes were no longer NP-associated.

A majority of NPs pass the LNs around the subcapsular sinus and arrive in the spleen after entering the blood circulation, irrespective of administration route. Thus, NP association with all cell types in the spleen was delayed relative to that in the LNs (Figure 2b), although the kinetics of splenic targeting via these two routes were similar (Figure 2b). For all cell populations in the spleen, i.d. administration led to equal or greater cell targeting compared to i.m. (Figure 2b), consistent with the greater bioavailability (Figure 2a). As in the LNs, NP distribution in the spleen after 24 h was dominated by the association with B cells (i.d.: 6±3% vs. i.m.: 3.2±0.7) and T cells (i.d.: 7±1% vs. i.m.: 5±2%) due to their dominance in overall cell numbers. Among the other cell types, monocytes, medullary/red pulp macrophages, and granulocytes were particularly well-targeted in the spleen (Figure 2b, panels 8–10).

To better understand the relative importance of the administration route for targeting each cell type, we built a statistical regression model and partitioned the entire cellular distribution data set into a hierarchical binary tree [24] according to the significance of each subset, comparing the route of administration (Figure S5). Within the LNs, the model demonstrated that route of administration is significant only for the sentinel (i.e., popliteal and axillary) LNs (sentinel draining LNs) for NP association, confirming the physiology and anatomy of lymphatic drainage from the skin (Figure S5). Accordingly, the route of administration did matter as much for particular cell targeting in the downstream LNs (i.e.,brachial and inguinal). To further confirm the importance of the route of administration on the specific cellular compartments targeted, we formulated an alternate regression model in which the factor of the administration was neglected, and we compared it with the full model for each cell type (Figure 2c). The importance of the i.d. administration, quantified as the log-likelihood ratio of the alternate model over the full model, was pronounced within the overall leukocyte population, the DC subpopulations, monocytes and granulocytes, while it was indifferent for the medullary macrophages, most likely due to their spatial location in the secondary organs. This, in conjunction with blood bioavailability results (Figure 2a), emphasized that superiority of the i.d. route over i.m. for targeting NPs to the LNs, blood, and spleen. Together, these findings highlight the significance of the i.d. route for effective delivery to immature and cross-presenting APCs in sentinel LNs.

### Nanoparticle trafficking to the LN relies on lymphatic drainage

**Figure 3:**
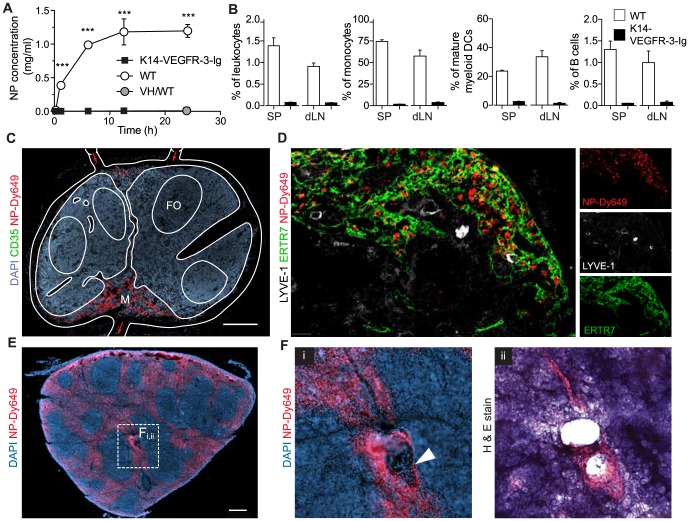
Lymphatic drainage is required for nanoparticle targeting of the lymph node and spleen after i.d. administration. (**a**) Bioavailability of Dy649-nanoparticles (NPs) in the blood compartment after i.d. administration in mice that lack peripheral lymphatics (K14-VEGR-3-Ig) and their wild type littermates. VH: vehicle control (non-fluorescently labeled NPs). Two-way repeated measures ANOVA followed by Bonferroni post test. (**b**) Comparison of NP^+^ association as assessed by flow cytometry in the brachial lymph node (LN) and the spleen 24 h post-i.d. administration. n = 4 *p≤0.05, ***p≤0.005. (**c**) 9 µm thick section of a Dy649-NP (red) draining wild type popliteal LN stained with nuclei (DAPI, blue). Scale bar, 200 µm. (**d**) 9 µm thick section of the NP-Dy649 (red) draining wild type brachial LN 12 h after i.d. administration, stained for lymphatic endothelium (LYVE-1, green), the T cell zone stroma (ERTR7, white). Scale bar, 40 µm. (**e**) 40 µm section of the wild type anterior spleen stained with DAPI (blue) and NP-Dy649 (red) shows NP accumulation in the red pulp (RP) and the marginal zone (MZ), as well as surrounding the B cell follicles (FO). Scale bar, 300 µm. (**f**) Enlarged region of the central arteriole of the spleen (white filled arrow) (***i***) immunofluorescence image (NP-Dy649, red and DAPI, blue). (***ii***) Hematoxylin & eosin staining of the same section of the spleen; dark blue FO, purple red pulp and pink blood vessels. Scale bar, 100 µm.

The early (≤6 h) association of NPs with LN-resident leukocytes suggests that lymphatic drainage from the administration site is a key determinant in uptake and distribution. To confirm this supposition, we inoculated i.d. the same amount of fluorescently labeled NPs in transgenic mice that lack peripheral lymphatics in the dermis. NPs injected into K14-VEGFR-3-Ig mice, which lack dermal lymphatic capillaries due to soluble VEGFR-3 secretion by keratinocytes [22], [45], were not present in the blood even 24 h after i.d. administration (Figure 3a). We observed minimal NP association in the draining LN and the spleen, as determined by measurements on leukocytes, monocytes, DCs and B cells (Figure 3b), among other compartments (data not shown). Therefore, drainage via the dermal lymphatics is essential to target cells in secondary lymphoid organs or blood after i.d. administration.

Upon entry into the initial lymphatics, we observed that NPs entered the LN from the afferent lymphatics as expected. However, rather than remaining in the subcapsular sinus and bypassing the LN interior (Figure 3c, red arrows), a substantial amount of NPs were seen entering the conduits to the medulla (Figure 3c, M), diverting from the B cell follicle (FO) or cortex regions. When stained for the stromal cell marker ERTR-7 and the lymphatic marker LYVE-1, which indicate the architectural pattern of drainage within the LN [46], the fluorescent NPs were mainly confined within the LYVE-1^+^ structures of the lymphatics (Figure 3d) and populated the LN medulla. In the spleen after 24 h, NPs had entered the organ from the central arteriole (Figure 3e and f, white arrowhead) and were found to populate the entire red pulp, reaching the marginal zone without penetrating the B cell follicles of the white pulp (Figure 3e and f), and were retained in the subcapsular space (Figure 3e).

These results demonstrate that the early cell association of the NPs after i.d. injection could be attributed to the initial drainage of NPs from the dermis into the lymphatics, and that the cell distribution within the LN was consistent with the cells that populate the medulla of LNs. For those NPs that entered the systemic circulation, the cell distribution within the spleen was consistent with entry from the central arteriole [47]. This anatomical distribution is consistent with the flow cytometric analysis of cell subset associations presented in Fig. 1 and 2b: namely, immature DCs and medullary macrophages are resident subpopulations, located in the medulla of LNs and around the marginal zone (MZ) in the spleen [47]; monocytes are predominantly resident in the bone marrow, but are also resident in the subcapsular red pulp of the spleen, from where they are deployed under inflammatory conditions [48]. Taken together, i.d. administration enhances NP association with monocytes and to an extent with antigen-presenting populations within the LN and spleen, following the pattern of lymphatic drainage.

### Intracellular vs. extracellular association of nanoparticles

**Figure 4:**
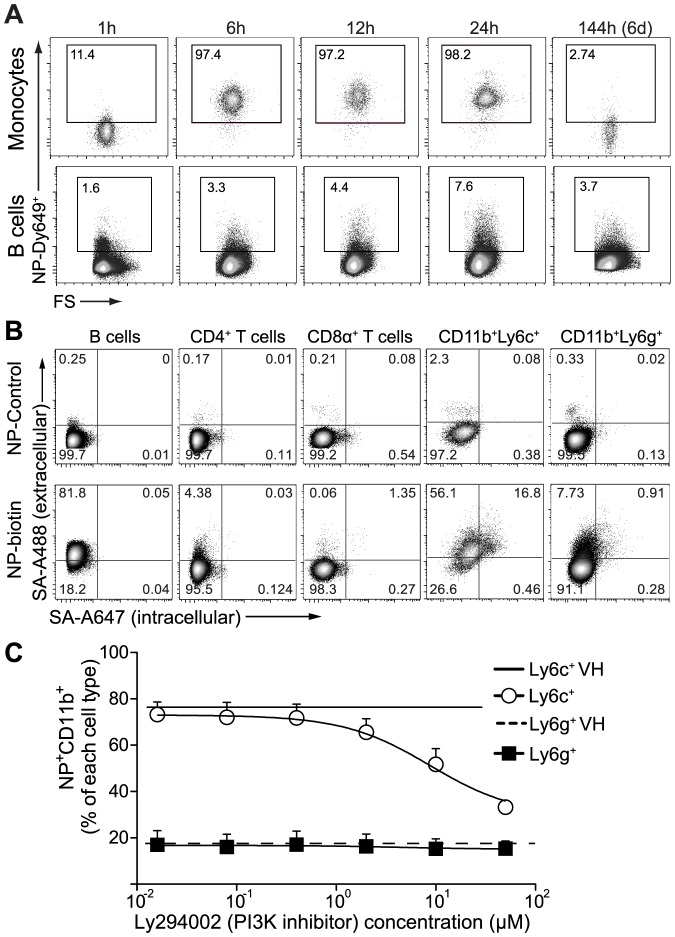
Monocytes internalize nanoparticles via macropinocytosis while B and T cell associate externally. (**a**) Representative flow cytometry plots of *in vivo* NP-Dy649^+^ uptake kinetics after intradermal administration: monocytes (CD11b^+^GR1^mid^SSC^low^F4/80^+^) and B cells (B220^+^) in the spleen. (**b**) Characteristic flow cytometry plots of biotinylated nanoparticle (NP-biotin) association with splenic (B, CD4, and CD8 cells) and bone marrow (CD11b^+^Ly6c^+^ and CD11b^+^Ly6g^+^) cells after 12 h incubation *in vitro*. To distinguish surface-associated- from internalized-NPs, cells were incubated before permeabilization with streptavidin-A488 (for extracellular association) and after permeabilization with streptavidin-A647 (for intracellular uptake). (**c**) Percentage of fluorescently labeled NPs (NP-Dy649) taken up by bone marrow cells as a function of the PI3K inhibitor (Ly294002) concentration. Bone marrow cells were incubated with increasing concentrations of Ly294002 (maximum 50 µM) for 45′ prior to the addition of NP-Dy649 for 12 h. Cells were subsequently stained and analyzed by flow cytometry. Open circles: CD11b^+^Ly6c^+^, filled squares: CD11b^+^Ly6g^+^, continuous line: vehicle control (VH, DMSO) for CD11b^+^Ly6c^+^, dashed line: VH for CD11b^+^Ly6g^+^.

In the *in vivo* kinetic study described above, we observed by flow cytometry that over the time course of observation, monocytes and granulocytes consistently displayed a distinct population shift, while B cells and T cells exhibited only a partial increase in fluorescence in NP-Dy649 channel with the main population not shifting (fluorescence for monocytes and B cells shown in Figure 4a). As we have previously shown that NPs are taken up by DCs via macropinocytosis [8], we asked whether monocytes, granulocytes, B cells, and T cells that associated with NPs *in vivo* followed the same mode of endocytosis. Specifically, using a multi-step extra- and intra-cellular streptavidin staining with two different fluorophores, we asked whether biotinylated NPs incubated with splenocytes *in vitro* were located extracellularly or intracellularly. We noted that B and T cells displayed a predominant shift only in the fluorescent channel related to the extracellular staining (Figure 4b, corresponding to double positive for streptavidin AF488 as well as AF647). On the other hand, NPs were found both externally and internally in the monocytic, while less amount of fluorescence was observed to co-localize within the granulocytic compartments. This confirmed that NPs were indeed endocytosed by monocytes and granulocytes (Figure 4b), while they stay predominantly on the exterior of T and B cells, associated with the plasma membrane. Surface association of lipid-based nanoparticles with T cells has been observed by others, mediated for example by cell-surface thiols from unpaired cysteine residues [49].

To further determine whether NPs were macropinocytosed by monocytes and granulocytes, we performed uptake studies of NPs using a phosphatidylinositol 3-kinase inhibitor of macropinocytosis (LY249004), which we have previously shown to block macropinocytosis in DCs [8]. Macropinocytosis was reduced in an inhibitor concentration-dependent manner (Figure 4c). We observed that CD11b^+^Ly6c^+^ monocytic myeloid cells took up fewer NPs, while CD11b^+^Ly6c^mid^Ly6g^+^ polymorphonuclear (Figure 4c) as well as B and T cells (data not shown) were not affected by LY249004 treatment. Thus, the biodistribution of NPs to detailed cell subsets also could be further separated into NP-internalizing and NP-associating cell types, a factor that determines the fate of the delivered payload and its effects on the cells [11].

### Nanoparticles target MDSCs in secondary lymphoid organs and tumors

Based on the observation that NPs were found in nearly all cells of the monocytic compartment (Figure 1b), and given that monocytes play a pivotal role in both inflammatory (as in myocarditis) [48] and immunosuppressive [50], [51] phenotypes (e.g., myeloid derived suppressor cells, MDSCs), we reasoned that the i.d. route of administration of NPs could be used to target immunosuppressive monocytes with therapeutic applications in mind. Cells of the monocytic origin, phenotypically characterized as CD11b^+^Ly6g^mid^Ly6c^+^ (or CD11b^+^GR1^+^SSC^low^F4/80^+^), derive from the bone marrow. They populate the blood compartment, accumulate in chronic inflammatory sites such as the tumor microenvironment [52], and traffic to secondary lymphoid organs, where, depending on the appropriate context, they exert their suppressive function. It has been shown that tumors can elicit significant amounts of MDSCs in the spleen [51] and in the tumor microenvironment [50], [53] in humans and mice. As MDSCs can suppress self or induced anti-tumoral responses in the microenvironment by secreting a plethora of biomolecules that either block T cell activation and proliferation or act upon DCs and macrophages to bias them towards tolerance-inducing APCs [54], MDSCs are significant targets for immunomodulation. Therapeutically, it was recently shown that systemic delivery of nanoparticles leads to selective uptake by inflammatory monocytic populations accumulating in atherosclerotic lesions, which can be reversed in mice by delivering CCR2-silencing short interfering RNA [55], [56].

**Figure 5:**
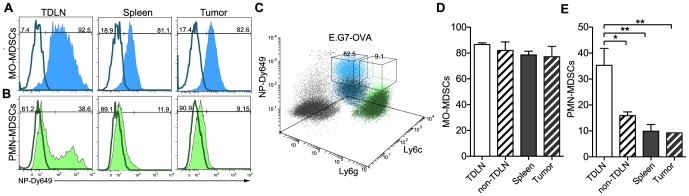
Nanoparticles are taken up by MDSCs in draining nodes, spleen and tumor. Mice were inoculated subcutaneously with 10^6^ E.G7-OVA thymoma cells underneath the left shoulder blade (dorsoanterior left lateral side). After tumors reached 100 mm^3^, mice were injected with Dy649-labeled nanoparticles (NPs). Flow cytometry plots illustrating targeting of (**a**) monocytic (MO) MDSCs and (**b**) polymorphonuclear (PMN) MDSCs in the tumor draining lymph node (TDLN), the spleen and the tumors. (**c**) Three-dimensional flow-cytometry representation of the MDSC compartment (MO and PMN) of the tumor. Comparison between different organs of interest of the (**d**) MO-MDSCs and (**e**) PMN-MDSCs subpopulation accumulating NPs. One-way ANOVA followed by Bonferroni post test. n = 3 *p≤0.05, **p≤0.01. Tu: Tumor; Sp: Spleen.

To address whether our NPs could be used to target MDSCs in tumor models, we used E.G7-OVA or B16-F10 cell lines grafted into immune competent C57Bl/6 mice. When tumors reached approximately 100 mm^2^, mice were administered i.d. fluorescently labeled NPs. 12 h after administration, we assessed the presence of MDSCs and their association with NPs in the tumor microenvironment, LNs and the spleen by flow cytometry (Figure 5a–c for E.G7-OVA tumors and Figure S6 for B16-F10 tumors). Interestingly, the majority of the monocytic MDSCs (CD11b^+^Ly6g^−^Ly6c^+^) in the tumor-draining LN (TDLN) (87±1%), spleen (79±3%) and tumor microenvironment (77±8%) were NP^+^ (Figure 5a,d). The fact that monocytes continuously traffic from the secondary lymphoid organs to the site of inflammation [57] is consistent with our measurements of similar frequencies of NP^+^ monocytic MDSCs in these three organ sites. In contrast, the granulocytic MDSCs (CD11b^+^Ly6g^+^Ly6c^mid^) populating the TDLN displayed higher association (35±7%) than the non-TDLN (16±2%), spleen (10±3%) and tumor (9±3%) (Figure 5a,e). This further highlights the importance of lymphatic drainage to target not only circulating monocytic MDSCs but also to focus targeting of polymorphonuclear MDSCs in TDLNs. With this data, we conclude that NPs delivered through the i.d. route efficiently target local and systemic MDSCs by exploiting lymphatic drainage.

### Conclusions

Understanding the trafficking distribution of nanoparticles to specific immune cell populations in the secondary lymphoid tissues and blood filtering organs is of high importance, especially when dealing with formulations that carry potent payloads, such as chemotherapeutics and adjuvants. Here, we showed that that fully synthetic 30 nm Pluronic-stabilized poly(propylene sulfide) NPs accumulate in the draining LN and spleen, but not in the liver. We also highlighted that lymphatic flow is crucial for NP drainage, both in the i.d. injection site and in the LN itself. At the level of the cellular population, we showed NPs localized to the myeloid compartment, consistent with their phagocytic ability, including DCs in the LN and the spleen, demonstrating a bias towards medullary macrophages and mature myeloid DCs. Of particular interest in vaccination, among the DCs efficiently targeted were CD8α^+^ DCs that are efficient at cross-presentation. Among the myeloid populations efficiently targeted were monocytes, both in the LN and spleen, as well as MDSCs in the tumor sites. Knowing the exact populations and timing of NP targeting will greatly accelerate the design of smarter, more efficient drug delivery and vaccine platforms while minimizing the risk of side-effects.

## Supporting Information

Figure S1 **Labeled nanoparticles were free of unconjugated dye.** (**a**) Nanoparticle conjugation scheme with Dy649-maleimide. (**b**) Characteristic analytical HPLC elution profiles of nanoparticles before and after dialysis. NP conjugated with Dy649-maleimide were injected in a size exclusion column (ID = 3 mm, L = 300 mm) packed with Sepharose® CL6B and analyzed in a fluorescent detector (excitation = 655 nm, emission = 685 nm).
(EPS)

Figure S2 **Gating strategy of the innate immune cell compartment.** Characteristic flow cytometry analysis of a spleen injected with control nanoparticles (black) or Dy649-nanoparticles (blue). Populations described within the manuscript are highlighted below. (a) Cross-presenting dendritic cells: CD11c^+^CD8α^+^; (b) medullary/red pulp macrophages (MØ): GR1^low^SSC^high^CD11b^+^F4/80^+^; (c) granulocytes: CD11b^+^GR1^high^SSC^high^; (d) monocytes: CD11b^+^GR1^mid^SSC^low^F4/80^+^; (e) mature myeloid DCs: CD11c^+^CD11b^+^I/A^b+^; (f) immature myeloid DCs: CD11c^+^CD11b^+^I/A^b−^; (g) immature lymphoid DCs: CD11c^+^CD11b^−^I/A^b−^.
(EPS)

Figure S3 **Gating strategy of the adaptive immune cell compartment.** Characteristic flow cytometry analysis of a spleen injected with control nanoparticles (black) or Dy649-nanoparticles (blue). (**a**) CD45^+^ cells were separated to (**b**) B cells and T cells based on the expression of CD3ε and B220, respectively. T cell gate was further split up to CD4^+^, (**c**) CD8α^+^ and double negative T cells. CD4^+^ cells were characterized by their CD25 expression; (**d**) CD25^+^ and (**e**) CD25^−^. Double negative T cells were separated based on (**f**) TCRγδ^+^ cells.
(EPS)

Figure S4 **Tissue and cell biodistribution 12 h after intradermal administration of virosomes show preferential accumulation in the liver.** Dy649-NHS labeled L-α-phosphatidylethanolamine was incorporated into the beta-propiolactone inactivated-, nucleocapsid removed-A/Singapore/6/86 influenza virus and injected intradermally into C57Bl/6 mice. After 12 h, heat maps show that virosomes (VSs) were found preferentially associated with (**a**) leukocytes (CD45^+^) in the liver (12±8%). (**b**) B cells: B220^+^, T cells: (CD3ε^+^ then CD4^+^CD25^+^, CD4^+^CD25^−^, CD8^+^), TCRγδ: CD3ε^+^CD4^−^CD8^−^ TCRγδ^+^, immature myeloid dendritic cells (DCs): CD11c^+^CD11b^+^I/A^b−^, immature lymphoid DCs: CD11c^+^CD11b^−^I/A^b−^. (**c**) granulocytes: CD11b^+^GR1^high^SSC^high^, monocytes: CD11b^+^GR1^low^SSC^low^F4/80^+^, mature myeloid DCs: CD11c^+^CD11b^+^I/A^b+^, CD11c^+^CD8α^+^I/A^b+^, CD11c^+^CD11b^−^I/A^b+^, medullary macrophages (MØ): CD11b^+^F4/80^+^. Draining lymph nodes are indicated by Ax: axillary, Br: brachial, In: inguinal, Po: popliteal; Sp: spleen, Bl: blood, Kd: kidneys, Li: liver, Lu: lungs. Heatmap color scales indicated on the right. Several leukocyte subsets displayed (a, b) low to medium levels (0–15%) or (c) high levels (up to 98%) of association with VSs.
(EPS)

Figure S5 **Sentinel lymph nodes and the spleen are most affected by the route of administration.** (**a**) Hierarchical binary tree of all NP+ cellular compartments from the cell compartment kinetic analysis, comparing all the draining lymphoid organ of interest and the route of administration. Analysis was performed in the statistical analysis package R, using the betatree regression model. (**b**) Schematic representation of the location of the secondary lymphoid organs draining the injection site. Sentinel nodes: axillary (Ax) and popliteal (Po); non-sentinel nodes: Brachial (Br) and Inguinal (In); Spleen (Sp).
(EPS)

Figure S6 **Nanoparticles naturally target MDSCs in tumor-draining lymph nodes, spleen and tumor.** Mice were inoculated with 10^6^ B16-F10 melanoma cells, and when tumor volumes reached 100 mm^3^, mice were injected intradermally with fluorescently labeled nanoparticles (NPs), 12 h after NP administration the spleen and tumor were harvested, stained and analyzed by flow cytometry. Histograms illustrating targeting of (a) monocytic (MO) MDSCs and (b) polymorphonuclear (PMN) MDSCs in the spleen and the tumors.
(EPS)
